# Infectious disease burden and antibiotic prescribing in primary care in Israel

**DOI:** 10.1186/s12941-018-0278-5

**Published:** 2018-06-09

**Authors:** Marcelo Low, Ronit Almog, Ran D. Balicer, Nicky Liberman, Raul Raz, Avi Peretz, Orna Nitzan

**Affiliations:** 1 Clalit Research Institute & Chief Physician’s Office, Clalit Health Services, Tel Aviv, Israel; 2 School of Public Health, University of Haifa, Haifa, Israel; 3 Rambam Medical Center, Haifa, Israel; 4 Ben-Gurion University of the Negev, Beer-Sheva, Israel; 5 Clalit Health Services, Community Medicine Division, Tel-Aviv, Israel; 6 Rappaport Faculty of Medicine, Technion, Haifa, Israel; 7 Clinical Microbiology Laboratory, The Baruch Padeh Medical Center Poriya, Tiberias, Israel; 8 The Faculty of Medicine in the Galilee, Bar Ilan University, Zefat, Israel; 9 Infectious Disease Unit, Poriya Medical Center, Tiberias, Israel

**Keywords:** Infectious diseases, Antibiotics, Prescriptions, Burden, Outpatients, Primary care

## Abstract

**Background:**

Antibiotics are frequently prescribed at many of the visits to primary care clinics, often for conditions for which they provide no benefit, including viral respiratory tract infections.

**Objectives:**

The aim was to evaluate primary care visits due to infectious diseases, and to estimate antibiotic prescribing and antibiotic dispensing by pharmacies.

**Methods:**

Diagnosis of infectious disease, antibiotic prescribing and dispensing data at the individual patient level were extracted for 2015 from Clalit Health Services’ electronic medical records and linked to determine the condition for which the antimicrobial was prescribed.

**Results:**

There were 6.6 million visits due to infections, representing 22% of all primary care visits. The most common events were upper respiratory tract infections (38%) and pharyngitis (10%). Highest prescription rates were for urinary tract infections (80%), otitis media (64%), pharyngitis (71%), sinusitis (63%), and lower respiratory tract infections (76%). The highest rates of undispensed prescriptions were for acute gastroenteritis, urinary tract infections, and pharyngitis (24, 23, and 16%, respectively).

**Conclusions:**

Infectious diseases constitute a heavy burden on primary care, with overprescribing of antibiotics. Intervention to reduce unwarranted antibiotic use is needed. In pediatric care, interventions should focus on better controlling antibiotic consumption and encouraging adherence to guidelines for upper respiratory tract infections, pharyngitis, and otitis media. In adults interventions should aim to monitor antibiotic prescribing for upper respiratory tract infections and improve adherence to guidelines for urinary tract infections.

**Electronic supplementary material:**

The online version of this article (10.1186/s12941-018-0278-5) contains supplementary material, which is available to authorized users.

## Background

A decline in morbidity and mortality from infectious diseases was witnessed during the twentieth century due to antibiotics implementation, improvements in hygiene, sanitation, and implementation of routine childhood vaccination programs [1]. Despite this, infectious diseases remain the most common reason for primary care visits, which may constitute about a third of primary care consultations, with the leading infections being respiratory tract, followed by skin and urinary tract infections (UTI) [2–6].

In many of the visits at primary care clinics an antibiotic is prescribed, often for conditions for which they provide no benefit, including viral respiratory tract infections [1, 7]. Antibiotic prescribing in primary care steadily increased in developed countries until the 1990s, at which point it leveled off and then declined. However, in the past few years, prescribing rates may be increasing again [8]. Primary care is responsible for 80% of the antibiotics used worldwide, with an estimated 20–50% of use being deemed inappropriate [9, 10]. Different studies found that in up to 40% of visits at primary care clinics an antibiotic is prescribed, most commonly for upper respiratory tract infection (URTI), with broad spectrum antibiotics constituting up to half of the prescriptions [11].

Antibiotic use in the community plays an important role in predisposing antibiotic resistance [12, 13]. A meta-analysis studying the effects of antibiotic prescribing in primary care on antimicrobial resistance found that individuals prescribed an antibiotic for a respiratory or urinary tract infection are at increased risk for developing bacterial resistance to the antibiotic they received, with the effect persisting for up to 12 months [14]. Institutional, national, and international initiatives encouraging decreased antibiotic prescribing, by using guidelines and educational programs, have reduced antibiotic dispensing in some cases, with an effect of decreasing occurrence of antibiotic resistance [15–19].

In a prior study we found an increase in outpatient broad spectrum antibiotic consumption in Israel during the last decade, primarily among the adult population [20]. Currently, there are no published data from Israel linking the incidence of primary care visits for infectious causes, antimicrobial prescribing, and antimicrobial dispensing. Such data is crucial for understanding the reasons for the observed trends in antibiotic consumption and for planning interventional programs aimed at reducing antibiotic prescribing in the community.

The aim of this study was to evaluate the incidence of primary care visits due to infectious diseases in both children and adults, and to estimate the rate of antibiotics prescribed during these visits.

## Methods

### Study design and population

This cohort study included all insured members of Clalit Health Services (CHS) who have 12 consecutive months of membership or birth or death during 2015. CHS is the largest not-for-profit health fund in Israel, covering about 53% of the Israeli population, providing an extensive network with 14 hospitals, more than 1400 primary and specialized clinics around Israel. We excluded temporary residents or patients who were not identified as Israeli citizens. CHS insures members of all ages, every ethnic group, and all socioeconomic backgrounds, across the entire country. The study was approved by the Clalit Helsinki Ethics Committee.

### Database and study variables

Primary diagnosis data for common infectious diseases from outpatient visits during 2015 were extracted from electronic medical records. Diagnosis as coded by primary care physicians. Prescribing and dispensing data at the individual (de-identified) patient level were linked and extracted from a common database. Age at first day of event was categorized in the following groups: 0–2, 3–8, 19–44, 45–64, and 65+ years.

The number of specific disease episodes per 1000 persons was calculated. An infectious disease **episode** was defined as all consecutive visits belonging to the same infection group up to an interval of 14 days between the visits. Each infection group was classified according to the list of International Classification of Diseases, Ninth Revision (ICD9) and International Classification of Primary Care codes (ICPC) chosen by a clinician’s consensus group (see Additional file 1: Appendix).

### Statistical analysis

Descriptive statistics were used to examine outpatient visits determined to be due to infectious causes.

Infection-specific event rates and antibiotic prescription rates were calculated. We then analyzed the incidence of various common infectious diagnoses out of all the infectious events encountered in primary care during 2015, and the prescription rate of antibiotics for these infections. We used Chi square statistics and Cochrane–Armitage test for linear trend in order to evaluate whether infection rates by age groups are evenly distributed.

Analyses were performed using Statistical Packages for Social Sciences (SPSS) version 20, with *p* < 0.05 considered statistically significant.

## Results

In December 2015, there were 4,348,323 patients insured by Clalit Health Services in Israel. From January to December 2015, there were 6.6 million visits in outpatient clinics due to infectious causes, representing 22% of all primary care visits during that year. Upon exclusion of re-consultations for the same infectious episode, we established that 2.3 million patients visited outpatient clinics for 4.8 million different infectious events during this year.

**Fig. 1:**
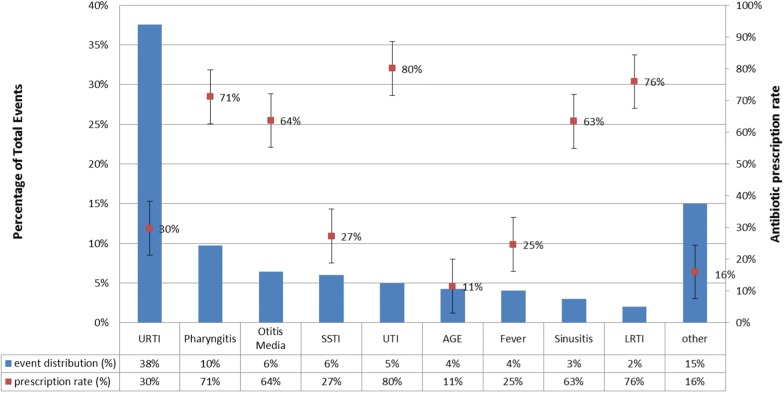
Event distribution and antibiotic prescription rate by infection type. *AGE* Acute Gastroenteritis, *LRTI* Lower Respiratory tract infections, *SSTI* Skin And Soft Tissue Infections, *URTI* Upper Respiratory tract infections, *UTI* Urinary Tract Infections

Upper respiratory tract infections (URTI) constitute 38% of all infectious events (Fig. 1). Pharyngitis was diagnosed in 10% of infectious events, followed by Conjunctivitis (7%), Acute Gastroenteritis (AGE) and Acute Otitis Media (AOM) in 6% of events each, Skin and Soft Tissue Infections (SSTI) in 5% of events, Urinary Tract Infections (UTI) and Fever in 4%, Sinusitis in 3%, and Lower Respiratory Tract Infections (LRTI)—pneumonia and bronchitis in 2% of events. Figure 1 also shows the antibiotic prescription rates for each infection, the highest rates of systemic antibiotics were prescribed for UTIs (80%), LRTI (76%), pharyngitis (71%), AOM (64%), and sinusitis in 63% of events. The lowest prescription rates were for URTI (30%), SSTI (27%), fever (25%), AGE (11%), and other infections in 16%.

**Fig. 2:**
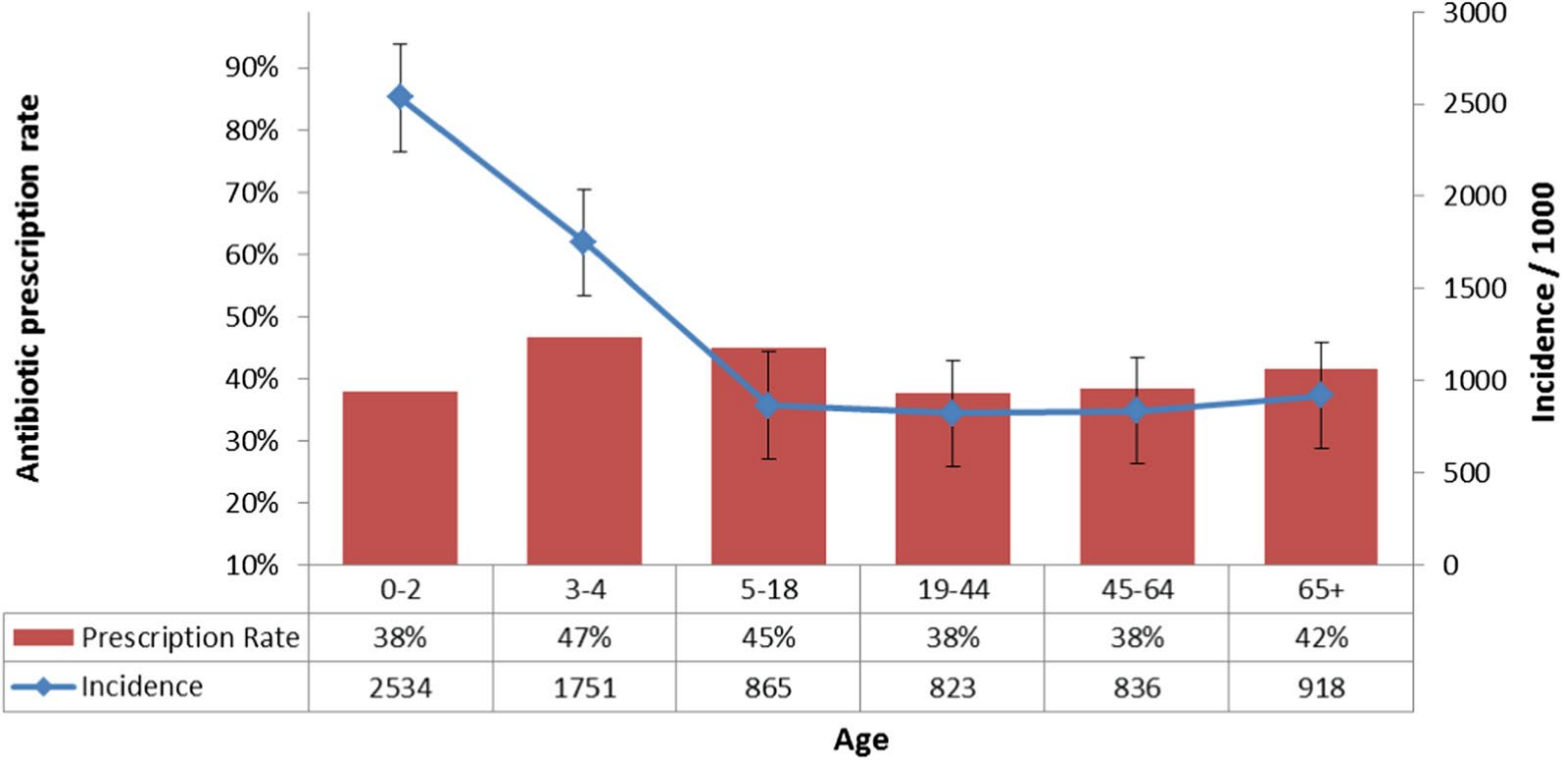
Incidence rates of all infections and antibiotic prescriptions by age

Examination of the incidence of all infectious events in different age groups (Fig. 2) revealed that the incidence of infections encountered in primary care was highest in young children with 2534 infectious events per 1000 children aged 0–2-years-old, decreasing in a linear trend (*p* < 0.01) to 1751 and 865 events per 1000 in children 3–4- and 5–18-years-of-age, respectively. The incidence rate of infectious diseases remains stable, around 800–900 events per 1000 people in late childhood and adult age groups.

The average overall antibiotic prescription rates among all age groups were 38–47% with no significant differences between the various disease groups.

**Fig. 3:**
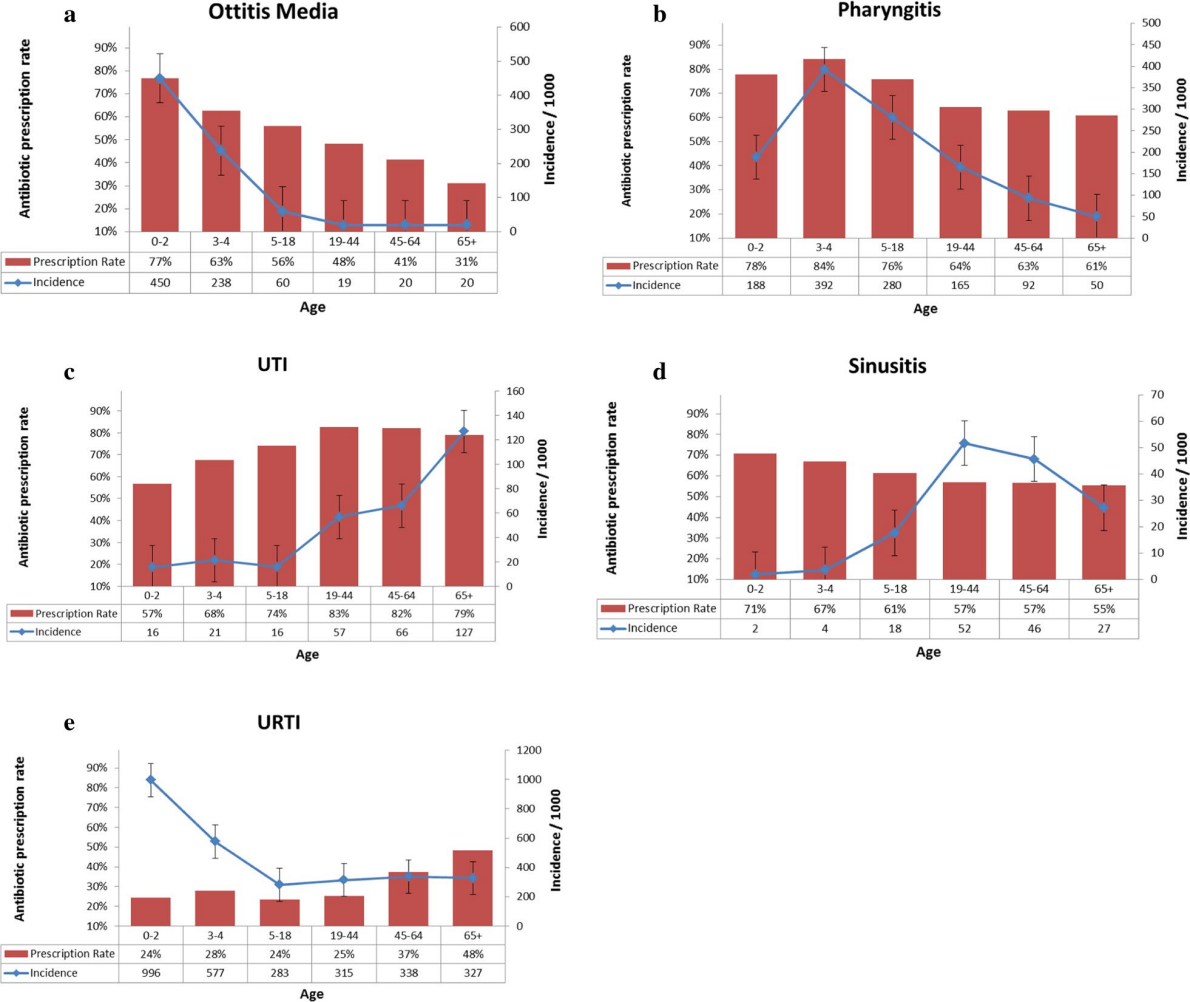
Incidence and antibiotic consumption rates for **a** otitis media, **b** pharyngitis, **c** UTI, **d** sinusitis, and **e** URTI by age

The incidence of AOM was highest among young children 0–2-years-old, 450 per 1000, and 238 cases per 1000 in children 3–4-years-old. The incidence rates for AOM sharply declined among the older age groups (*p* for trend < 0.05) (Fig. 3a). Also, a higher incidence rate among younger ages and a linear decline and lower incidence rates among adults were found in URTI events (Fig. 3e). In contrast, UTIs were encountered most frequently in older adults (127 events per 1000 in the > 65-year-old group, around 60 per 1000 in the 19–64-year-old group, to 16–21 events per 1000 in the youngest age group (*p* for trend < 0.01) (Fig. 3c). The incidence of sinusitis was highest (52 per 1000) in the 19–44-year-old group and decreased to 27 per 1000 in the 65+ age group. Sinusitis is rare among younger ages and more frequently diagnosed among late childhood, 5–18 years, and adults (Fig. 3d) (Chi square *p* < 0.01). Figure 3a–d also show prescription rates of antibiotics.

For sinusitis, prescription rates were around 50–60% among adults.

The highest rates of antibiotics prescribed for AOM were among young children (77% in 0–2-year-olds and 63% in 3–4-year-olds), and for pharyngitis in 3–4-year-olds (84%) (Chi square *p* < 0.01). High prescription rates for UTIs (80%) were found in adult age groups.

**Fig. 4:**
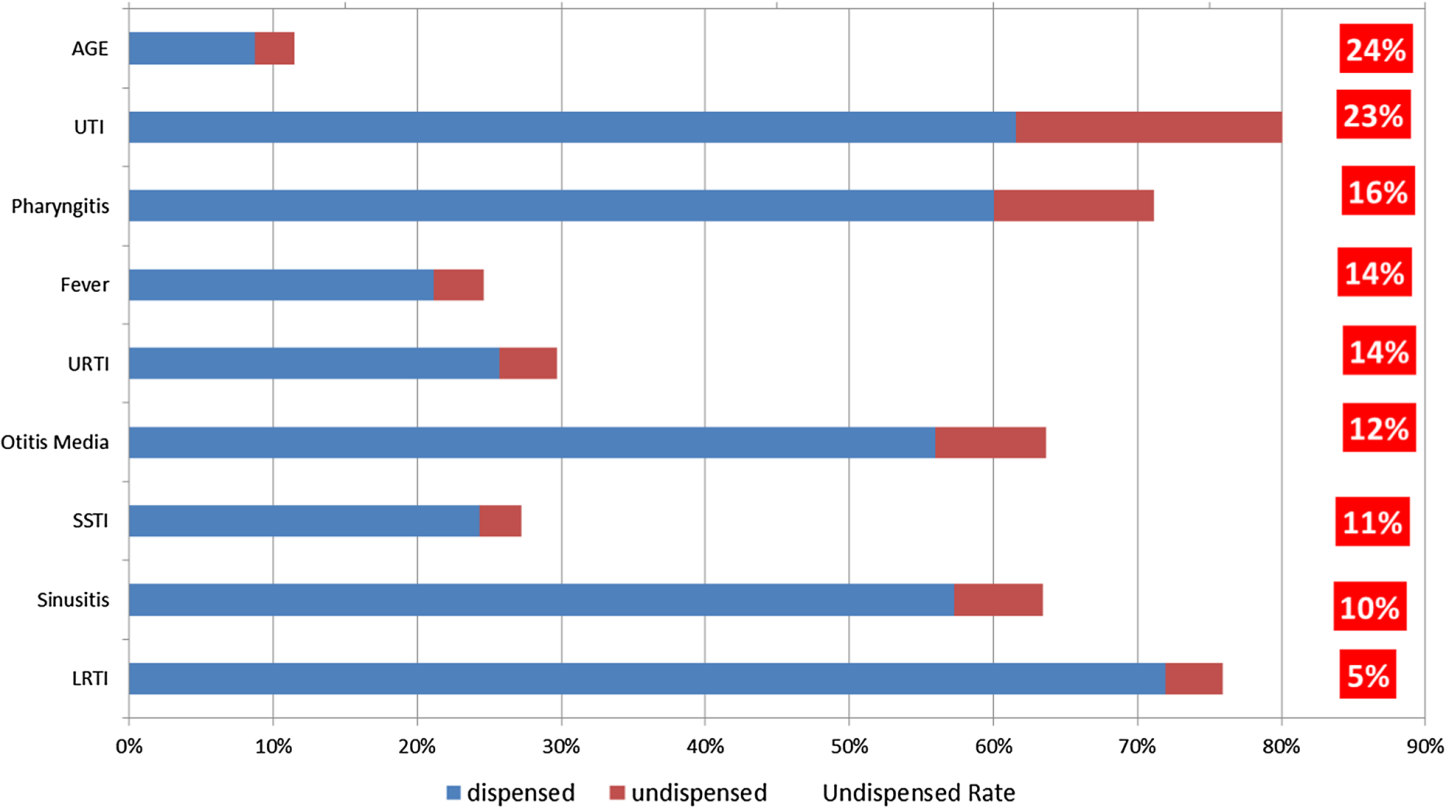
Undispensed antibiotic rate by infection type. *AGE* Acute Gastroenteritis, *LRTI* Lower Respiratory tract infections, *SSTI* Skin And Soft Tissue Infections, *URTI* Upper Respiratory tract infections, *UTI* Urinary Tract Infections

In assessing how many of the antibiotics prescribed by primary care physicians were actually dispensed by pharmacies, we found the highest rate of undispensed prescriptions for AGE (24%), UTI (23%), pharyngitis (16%), fever and URTI (14%), OM (12%), SSTI (11%), sinusitis (10%), and LRTI (5%) (Fig. 4).

## Discussion

Infections are the one of the most common reasons for primary care visits, entailing high costs and a portion of unnecessary antibiotic prescriptions [2, 10]. Our study contributes to current literature by uniquely combining data on primary care visits, diagnoses, and prescription and dispensing data from a large administrative and electronic health record data warehouse. Over-the-counter antibiotic purchase is illegal in Israel and therefore every prescription purchased by the CHS members is registered in the central administrative and electronic health record based data warehouse.

In this large cohort study, we found that 1 in 5 primary care visits are due to infectious causes, representing 6.6 million visits.

Our study may provide data to support stewardship programs focusing subgroups with higher likelihood of unwarranted use of antibiotics. The most frequent infections encountered, in 38% of infectious events, were URTIs. Other studies also found respiratory tract infections to be the leading cause for primary care visits, with many unnecessary reconsultations, and an estimated cost of 51.4 million dollars a year for visits due to acute cough in children alone [1, 21].

Our results show the overall infection incidence in the younger ages is very high, more than 2.5 events per year per child and decreasing to almost one event yearly for the whole population after 5 years-of-age. In approximately 38–47% of events among all age groups antibiotics were prescribed. This finding can be the result of two processes that are difficult to understand separately. One is that doctors can easily diagnose the viral etiology of those infections in younger ages; most of them are URTI’s and AOM. The other is the extra caution of doctors when writing a prescription. A Spanish study found that of all patients over the age of 14 attending clinics during 2007, 33.2% presented with an infectious disease, most commonly (50.4%) a respiratory tract infection [6].

The European Surveillance of Antimicrobial Consumption has found high and increasing outpatient antibiotic use in most European countries with high seasonal variation, suggesting unnecessary usage for viral infections [22].

Several of the common infections examined in this study, such as URTIs and sinusitis, primarily have viral etiologies [23]; however, we found that antibiotic prescription rates for these infections were fairly high. Israeli and international guidelines recommend administration of antibiotic therapy only for acute bacterial sinusitis, which is suspected if symptoms or signs are severe or persist for more than 10 days [24–26]. We found that 30% of patients with a diagnosis of a URTI were prescribed an antibiotic.

Although URTIs are mainly of viral etiology and local and international guidelines do not recommend prescribing antibiotics without the suspicion of a bacterial cause, they are still frequently prescribed [25, 27].

Other studies have also found overprescribing for sinusitis [28]. A cross-sectional study of a national database of outpatient visits in the US found that antibiotics were prescribed in 82.3 ± 2.6% of 18.7 million visits for acute rhinosinusitis, with ear nose and throat (ENT) specialists administering antibiotics less frequently but choosing broader spectrum antibiotics [29]. A study from Minnesota found a 69.2% prescription rate of antibiotics in a sample of patients with URTI in primary care [30]. Another study of outpatient antibiotic prescribing in veteran’s clinics in Oregon reported a 35.4% prescription rate for respiratory tract infections that did not influence patient outcomes [31].

Studying different age groups, we found that antibiotic prescription rates were high among all age groups with prescription rates of 40–50%. Focusing on four of the infections that seemed to constitute a large bulk of primary care visits with high prescription rates, we found that although the incidence of these infections varied, as expected, with age, antibiotics were prescribed in most cases and in all age groups. Although recent guidelines suggest a wait and see approach of follow-up and beginning antibiotic therapy only if the child worsens or fails to improve [32], our study’s findings of 63–77% prescription rates in children up to age four are consistent with a previous study’s high rates of antibiotic prescriptions for AOM in children [33]. A Dutch study found 40–50% prescription rates for AOM in children between the years 2002 and 2008 [34]. In pharyngitis, we found high antibiotic prescription rates (over 60%) in all age groups, including very young children, even though Infectious Disease Society of America (IDSA) guidelines do not recommend testing for group A streptococcus (GAS) in children under the age of 3, since the disorder is primarily a disease of children 5–15-years-of-age, and even in this age group, viruses are the most common cause of acute pharyngitis [35]. A recent Pakistani study found a 98.5% prescription rate for adults with pharyngitis while GAS was found by culture in only 4.4% of patients [36]. We found that between 75 and 90% of antibiotics prescribed by primary care physicians for all infections, excluding lower respiratory tract infections, are actually bought at the pharmacies. This might imply that many of the prescriptions are unnecessary, and thus the patients do not need to buy the antibiotic that was prescribed; also, physicians may over-perceive the need or desire of the patient to leave the office with a prescription.

The findings in our study may imply a need for improving the adherence to guidelines. Other studies have also found low adherence to guidelines—in a retrospective cohort study at an ambulatory facility practice in Pennsylvania, guideline adherence was 24% in patients with pharyngitis, 42% in acute sinusitis, 79% in URI, and 57% overall [37]. A German national survey found a rise in prescription rates from 2004 to 2006 in children and adolescents, specifically for tonsillitis, bronchitis, otitis media, and acute upper respiratory infections, due to low adherence to guidelines [38]. A prior Israeli study found a 35.6% adherence rate to guidelines for the treatment of uncomplicated UTIs in women [39].

The reasons for prescribing antibiotics discordantly with guidelines have been studied extensively. In a Canadian historical cohort it was shown that inappropriate antibiotic prescribing increased with time in practice and was also more frequent among foreign medical graduates and among primary care physicians with a high practice volume [40]. Some other studies concluded that a high frequency of antibiotic prescriptions may reflect a general disposition among general practitioners to give priority to maintaining good relations with their patients, as patients often expect to receive pharmacological treatment. A study performed in outpatient clinics in two counties in the south of Sweden demonstrated that the decision to prescribe antibiotics is dependent on the physician, the patient–physician relationship, the organization of primary care, as well as organizational culture.

How can we decrease the overprescribing of antibiotics and improve adherence to guidelines in primary care? Different studies have addressed these questions and found that educational programs, surveillance measures, and antimicrobial stewardship interventions can decrease antibiotic prescription rates in primary care [1]. A recent study found that a 1-h on-site clinician education session followed by 1 year of personalized audit and feedback, decreased antibiotic prescriptions for URTIs by 12.5% [19]. A large British trial of 68 general practices in Wales found that a multifaceted educational program decreased antibiotic dispensing rates by 4.2% [17]. The effectiveness of such large scale interventions in the Israeli primary care setting is yet to be thoroughly studied.

There are several limitations to consider in this study. The infectious events were defined according to the empirical evidence as coded by the primary care physicians and did not necessarily confirm their bacterial etiology. This requires a more conservative approach regarding the overprescribing rates. We measured rates of prescriptions not purchased in several infections. The highest ratios recorded were among AGE, UTI, and pharyngitis events; this may be related to delayed antibiotic prescriptions while awaiting the result of the sensitivity test. Much less common is to find unpurchased prescriptions in LRT infections because this condition is more often treated in hospitals than in the community. On the other hand, the un-purchased rates may be another estimate of unneeded prescriptions.

The population included for this study was large, and infection is a very common event, including all group ages, and was nationally representative, thereby minimizing selection biases.

## Conclusions

Infectious diseases constitute a heavy burden on primary care in Israel, with overprescribing of antibiotics. Intervention to reduce unwarranted antibiotic use is needed. In pediatric care, interventions should focus on better control of antibiotic consumption and encouraging adherence to guidelines for URTIs, pharyngitis, and otitis media. In adults interventions should aim to monitor antibiotic prescribing for URTIs and improve adherence to guidelines for UTIs. In an era of increasing antibiotic resistance, efforts should be focused on antibiotic stewardship in primary care.

## Additional file

**Additional file 1: Appendix.** Diagnostic conditions ICD9 classification and antibiotic recommendation reference.
