# Supplementary material for: Infectious disease burden and antibiotic prescribing in primary care in Israel
Source: Ann Clin Microbiol Antimicrob. 2018 Jun 9;17:26. doi: 10.1186/s12941-018-0278-5 (PMC5994243; doi:10.1186/s12941-018-0278-5)
Supplement: Supplementary file 1 — Additional file 1: Appendix. Diagnostic conditions ICD9 classification and antibiotic recommendation reference. [file 12941_2018_278_MOESM1_ESM.docx]

Appendix: Diagnostic Conditions ICD9 classification and Antibiotic recommendation reference

| Condition | ICD9/ICPC codes | Probability of Antibiotic treatment  (Guidelines reference) |
| --- | --- | --- |
| Upper Respiratory tract Infections – URTI | 033,460, 464- 467,472,474,476,477, 487,491,786.2 ,  R74, R77, R78, R80, R83 | Antibiotics not indicated  [1-3] |
| Pharyngitis | 034,462, 463,  R72 | Antibiotics may be indicated  [4,5] |
| Acute Gastro Enteritis – AGE | 001-009,  D70 | Antibiotics may be indicated  [6] |
| Acute Otitis Media – AOM | 381-386  H71,H74 | Antibiotics may be indicated  [7] |
| Skin and Soft Tissue Infections – SSTI | 566,604, 680-686,  R73, S10, S76, L70 | Antibiotics may be indicated  [7-9] |
| Urinary Tract Infections – UTI | 590,595,599  U70,U71 | Antibiotics almost always indicated  [10,11] |
| Fever | A03 | Antibiotics may be indicated  [3,12] |
| Sinusitis | 461, 473  R75 | Antibiotics may be indicated  [7,13] |
| Lower Respiratory tract infections – LRTI | 073.0, 480-486, 487.0  R81 | Antibiotics almost always indicated  [12,14] |

Reference List for Additional file

[1] Dowell SF, Schwartz B, Phillips WR. Appropriate use of antibiotics for URIs in children: Part II. Cough, pharyngitis and the common cold. The Pediatric URI Consensus Team. Am Fam Physician. 1998;58(6):1335-42, 1345.

[2] Tan T, Little P, Stokes T.. Antibiotic prescribing for self limiting respiratory tract infections in primary care: summary of NICE guidance. BMJ. 2008;337:a437.

[3] Wong DM, Blumberg DA, Lowe LG. Guidelines for the use of antibiotics in acute upper respiratory tract infections. Am Fam Physician. 2006;74(6):956-66.

[4] Randel A. IDSA Updates Guideline for Managing Group A Streptococcal Pharyngitis. Am Fam Physician. 2013;88(5):338-40.

[5] Shulman ST, Bisno AL, Clegg HW, Gerber MA, Kaplan EL, Lee G, et al. Clinical practice guideline for the diagnosis and management of group A streptococcal pharyngitis: 2012 update by the Infectious Diseases Society of America. Clin Infect Dis. 2012;55(10):1279-82.

[6] De BG, Hahn S, Borwick A. Antibiotic treatment for travellers' diarrhoea. Cochrane Database Syst Rev. 2000;(3):CD002242.

[7] Rosenfeld RM. Clinical Practice. Acute Sinusitis in Adults. N Engl J Med. 2016;375(10):962-70.

[8] Montravers P, Snauwaert A, Welsch C. Current guidelines and recommendations for the management of skin and soft tissue infections. Curr Opin Infect Dis. 2016;29(2):131-8.

[9] Stevens DL, Bisno AL, Chambers HF, Dellinger EP, Goldstein EJ, Gorbach SL, et al. Practice guidelines for the diagnosis and management of skin and soft tissue infections: 2014 update by the Infectious Diseases Society of America. Clin Infect Dis. 2014;59(2):e10-e52.

[10] Gupta K, Hooton TM, Naber KG, Wullt B, Colgan R, Miller LG, et al. International clinical practice guidelines for the treatment of acute uncomplicated cystitis and pyelonephritis in women: A 2010 update by the Infectious Diseases Society of America and the European Society for Microbiology and Infectious Diseases. Clin Infect Dis. 2011;52(5):e103-20.

[11] Roberts KB. Revised AAP Guideline on UTI in Febrile Infants and Young Children. Am Fam Physician. 2012;86(10):940-6.

[12] Mandell LA, Wunderink RG, Anzueto A, Bartlett JG, Campbell GD, Dean NC, et al. Infectious Diseases Society of America/American Thoracic Society consensus guidelines on the management of community-acquired pneumonia in adults. Clin Infect Dis. 2007;44(Suppl 2):S27-72.

[13] Smith SS, Kern RC, Chandra RK, Tan BK, Evans CT. Variations in antibiotic prescribing of acute rhinosinusitis in United States ambulatory settings. Otolaryngol Head Neck Surg. 2013;148(5):852-9.

[14] Bradley JS, Byington CL, Shah SS, Alverson B, Carter ER, Harrison C, et al. The management of community-acquired pneumonia in infants and children older than 3 months of age: clinical practice guidelines by the Pediatric Infectious Diseases Society and the Infectious Diseases Society of America. Clin Infect Dis. 2011;53(7):e25-e76.
